# Supplementary material for: Commensal and Pathogenic Members of the Dental Calculus Microbiome of Badia Pozzeveri Individuals from the 11th to 19th Centuries
Source: Genes (Basel). 2019 Apr 12;10(4):299. doi: 10.3390/genes10040299 (PMC6523138; doi:10.3390/genes10040299)
Supplement: Supplementary file 1 [file genes-10-00299-s001.zip › Supplementary-files.zip!/Supplementary Figure 1.docx]

**Figure S1.** Microbiome analyses of dental calculus samples compared to extraction blank control and soil samples. Panel A shows a PCoA of the beta-diversity (Bray-Curtis) of the dental calculus samples prior filtering the Operational Taxonomic Units (OTUs) identified in the blank control. Panel B shows a PCoA of the beta-diversity (Bray-Curtis) of the dental calculus samples prior and after filtering the OTUs identified in the extraction blank and soil samples. Panel C shows the percentage of OTUs at the phylum level for the dental calculus samples prior and after filtering the OTUs identified in the extraction blank and soil samples, as well as those identified in the mentioned controls.
