# Supplementary material for: Commensal and Pathogenic Members of the Dental Calculus Microbiome of Badia Pozzeveri Individuals from the 11th to 19th Centuries
Source: Genes (Basel). 2019 Apr 12;10(4):299. doi: 10.3390/genes10040299 (PMC6523138; doi:10.3390/genes10040299)
Supplement: Supplementary file 1 [file genes-10-00299-s001.zip › Supplementary-files.zip!/Supplementary Table 1_Read number.docx]

**Supplementary Table 1.** Number of sequences per sample prior- and post-filtering Operational Taxonomic Units (OTUs) identified in the soil samples from the archeological sites, as well as the extraction blank control.

| **Sample** | **Number of sequences**  **prior filtering** | **Number of sequences**  **post filtering** |
| --- | --- | --- |
| USK2170 | 39,831 | 3,602 |
| USK2297 | 60,566 | 6,689 |
| USK2299 | 40,109 | 5,527 |
| USK2339 | 23,041 | 5,259 |
| USK2340 | 42,589 | 5,968 |
| USK2379 | 58,441 | 9,119 |
| USK2383 | 33,771 | 3,461 |
| USK2399 | 49,552 | 5,079 |
| USK2776 | 62,850 | 6,510 |
| USK3160 | 52,227 | 5,486 |
| USK3219 | 72,919 | 7,060 |
| USK3391 | 49,532 | 8,237 |
| USK3395 | 73,562 | 3,053 |
| USK3417 | 84,459 | 4,805 |
| USK3641 | 90,232 | 6,761 |
| USK3653 | 61,603 | 3,618 |
| USK3743 | 77,841 | 11,167 |
| USK3746 | 65,652 | 3,934 |
| USK4821 | 50,523 | 16,647 |
| USK6015 | 52,251 | 7,604 |
| USK6054 | 59,299 | 3,406 |
| USK6065 | 57,608 | 8,403 |
| USK6071 | 77,750 | 2,388 |
| USK6072 | 92,922 | 8,756 |
| USK6073 | 69,981 | 4,034 |
| USK6082 | 79,888 | 4,287 |
| Periodontitis-Plaque-1 | 42,160 | 34,313 |
| Periodontitis-Plaque-2 | 41,381 | 27,701 |
| Periodontitis-Plaque-3 | 52,871 | 50,929 |
| Periodontitis-Plaque-4 | 46,761 | 38,803 |
| Saliva-1 | 8,011 | 7,727 |
| Saliva-2 | 5,103 | 5,000 |
| Saliva-3 | 5,874 | 5,769 |
| Saliva-4 | 4,947 | 4,683 |
| Subgingival-1 | 3,738 | 3,580 |
| Subgingival-2 | 5,226 | 4,171 |
| Subgingival-3 | 5,836 | 4,533 |
| Subgingival-4 | 4,738 | 4,287 |
| Supragingival-1 | 6,742 | 5,474 |
| Supragingival-2 | 8,556 | 6,565 |
| Supragingival-3 | 4,970 | 3,945 |
| Supragingival-4 | 5,271 | 4,485 |
| Soil-1 | 52,111 | NA |
| Soil-2 | 34,786 | NA |
| Blank | 3,824 | NA |
