# Supplementary material for: Commensal and Pathogenic Members of the Dental Calculus Microbiome of Badia Pozzeveri Individuals from the 11th to 19th Centuries
Source: Genes (Basel). 2019 Apr 12;10(4):299. doi: 10.3390/genes10040299 (PMC6523138; doi:10.3390/genes10040299)
Supplement: Supplementary file 1 [file genes-10-00299-s001.zip › Supplementary-files.zip!/Supplementary Table 2.docx]

**Supplementary Table 2.** Additional information of the public sequence data included for comparisons with the dental calculus samples is shown. Information include 16S rRNA gene region sequenced, sequencing platform, sex, sample type, health status, collection country and study.

| **Accession number** | **16S rRNA gene**  **region sequenced** | **Sequencing**  **platform** |  | **Sex** | **Sample type** | **Health status** | **Collection country** | **Study** |
| --- | --- | --- | --- | --- | --- | --- | --- | --- |
| SRS017345 | V3-V5 | Titanium |  | male | Saliva | Healthy | United States | NIH Human Microbiome Consortium |
| SRS013228 | V3-V5 | Titanium |  | male | Saliva | Healthy | United States | NIH Human Microbiome Consortium |
| SRS013185 | V3-V5 | Titanium |  | male | Saliva | Healthy | United States | NIH Human Microbiome Consortium |
| SRS064596 | V3-V5 | Titanium |  | male | Saliva | Healthy | United States | NIH Human Microbiome Consortium |
|  |  |  |  |  |  |  |  |  |
| SRS021816 | V3-V5 | Titanium |  | female | Subgingival plaque | Healthy | United States | NIH Human Microbiome Consortium |
| SRS022119 | V3-V5 | Titanium |  | female | Subgingival plaque | Healthy | United States | NIH Human Microbiome Consortium |
| SRX020546 | V3-V5 | Titanium |  | female | Subgingival plaque | Healthy | United States | NIH Human Microbiome Consortium |
| SRS023328 | V3-V5 | Titanium |  | female | Subgingival plaque | Healthy | United States | NIH Human Microbiome Consortium |
|  |  |  |  |  |  |  |  |  |
| SRS013252 | V3-V5 | Titanium |  | male | Supragingival plaque | Healthy | United States | NIH Human Microbiome Consortium |
| SRS042272 | V3-V5 | FLX |  | female | Supragingival plaque | Healthy | United States | NIH Human Microbiome Consortium |
| SRS020893 | V3-V5 | Titanium |  | male | Supragingival plaque | Healthy | United States | NIH Human Microbiome Consortium |
| SRS022315 | V3-V5 | Titanium |  | female | Supragingival plaque | Healthy | United States | NIH Human Microbiome Consortium |
|  |  |  |  |  |  |  |  |  |
| SRR3503166 | V4 | Illumina MiSeq |  | male | Subgingival plaque | Periodontitis | United States | [24] |
| SRR3503259 | V4 | Illumina MiSeq |  | male | Subgingival plaque | Periodontitis | United States | [24] |
| SRR3503292 | V4 | Illumina MiSeq |  | female | Subgingival plaque | Periodontitis | United States | [24] |
| SRR3503326 | V4 | Illumina MiSeq |  | female | Subgingival plaque | Periodontitis | United States | [24] |
